# Factors influencing the uptake and utilization of cervical cancer screening services among women attending public health centers in Addis Ababa, Ethiopia: mixed methods study

**DOI:** 10.1186/s12905-023-02850-x

**Published:** 2024-01-02

**Authors:** Kemal Hussein, Gilbert Kokwaro, Francis Wafula, Getnet Mitike Kassie

**Affiliations:** 1 Institute of Healthcare Management, Strathmore University, Nairobi, Kenya; 2 International Institute for Primary Healthcare – Ethiopia (IPHC-E), Addis Ababa, Ethiopia

**Keywords:** Cervical cancer, Screening, Uptake, Utilization, Women

## Abstract

**Background:**

Cervical cancer is the second cause of cancer deaths among Ethiopian women. Despite multifaceted government efforts, the uptake and utilization of cervical cancer screening remain very low. This study aimed to assess factors influencing the uptake and utilization of cervical cancer screening at public health centers in Addis Ababa.

**Methods:**

A convergent parallel mixed-method study was employed to collect data through eight focus group discussions with 66 women purposively recruited from outpatient clinics, and cross-sectional face-to-face exit interviews with 80 women attending cervical cancer clinics in four high-patient volume health centers. The group interviews were tape-recorded, transcribed in Amharic, translated into English, and a thematic analysis approach was used in the analysis. Exit interview data were collected using a structured questionnaire in the Open Data Kit tool on an android tablet. STATA version 17 was used for descriptive and inferential data analyses. Statistical significance was set at *p* < 0.05.

**Results:**

The majority of focus group discussion participants had lack of knowledge of cervical cancer and its screening services. The major barriers to the uptake of screening were inadequate public awareness, fear of the procedure, embarrassment, provider’s gender, lack of male partner support, and childcare. Women aged 40 years and above were 13.9 times more likely to utilize cervical cancer screening than those under 30 years (AOR = 13.85; 95% CI: 1.40, 136.74). There was a strong preference for a female provider (AOR = 7.07; 95% CI: 1.53, 32.75) among women screened after attending antiretroviral therapy clinics and those screened due to abnormal vaginal bleeding than women referred from family planning clinics (AOR = 6.87; 95% CI: 1.02, 46.44). Safety of screening was negatively associated with women aged 30–39 (AOR = 0.045; 95% CI: 0.003, 0.696), and those who attended primary education, and secondary education and above, (AOR = 0.016; 95% CI: 0.001, 0.262), and (AOR = 0.054; 95% CI: 0.004, 0.724), respectively.

**Conclusions:**

The study identified low public awareness, inadequate provider preference, safety concerns, and poor male partner support for cervical cancer screening. We recommend the decision-makers enhance public messages, maintain provider choices, ensure safety, and engage males to improve the uptake and utilization of cervical cancer screening.

**Supplementary Information:**

The online version contains supplementary material available at 10.1186/s12905-023-02850-x.

## Background

The 2017 WHO Cancer Resolution recognized cancer as an increasing public health issue globally [1]. There were 604, 127 new cervical cancer cases with 341,831 deaths globally in 2020 [2]. The fourth most frequent type of cancer among women worldwide is cancer of the cervix uteri [3]. Cervical cancer is the second most common female cancer in Africa, with 117,316 new cases and 76,745 deaths reported in 2020 [4], which showed no significant rise from the 76,400 deaths reported in 2018 [5]. Cervix uteri cancer accounted for 23.3% of all new cases of cancer in females in Sub-Saharan Africa (SSA) in 2020 [6]. Eastern Africa had the highest age-standardized incidence rate (ASIR), with 40 cases per 100,000 women in 2020 [2]. This is ten times higher than the WHO Global Strategy’s target incidence rate of less than four per 100,000 women [7]. Infection with the high-risk strain of the human papillomavirus (HPV) was estimated to affect 34% of SSA women [8].

In Ethiopia, the most common cancers for women were breast and cervical cancer, respectively [9, 10] with cervix uteri incidence of 7, 445 cases and 5, 338 deaths in 2020 [11]. An ASIR of 24.6 per 100,000 Ethiopian females was reported in 2019 [12]. The country had 36.9 million women aged 15 years and older who were at risk of developing cervical cancer [13]. In Addis Ababa, the most common cancers were breast (33%), and cervix uteri (13.4%) [10, 14].

Though cancer has become an overt public health challenge in Ethiopia it was not given priority partly due to the strong focus on communicable diseases in the context of limited resources [14]. This challenge was compounded by a low cervical cancer screening service utilization of 5.47% among Ethiopian women [15]. The Ethiopian Health Sector Transformation Plan II (HSTP II) aims to increase the cervical cancer screening of women aged 30–49 years from 5 to 40% [16]. The National Cancer Control Plan (NCCP) applies “screen and treat” in the same facility where feasible as recommended by the WHO [17]. Though the country put in place various policies, cervical cancer screening remains low coupled with high morbidity and mortality figures. We hypothesize that this is due to the demand-side challenges of screening services. In Addis Ababa, cervical cancer focal persons (nurses, midwives, or health officers) were in charge of the public health centers’ cervical cancer services, including public education, screening, and treatment. While the organization, human resources, and scope of cervical cancer services varied among private clinics/health centers. Additionally, taking into account the private facilities was discovered to be resource-intensive due to the large number of facilities. Therefore, this study sought to identify and address factors affecting the uptake and utilization of cervical cancer screening services among women attending public health centers in Addis Ababa.

## Materials and methods

### Study design and period

The study used a convergent parallel mixed-method study design to examine factors influencing the uptake (qualitative) and utilization (quantitative) of cervical cancer screening services. The qualitative (focus group discussions, FGDs) were considered to explore factors influencing the uptake (perception, personal, financial, and sociocultural barriers), while the quantitative (exit interviews) focused on describing barriers (perception, referral system, coordination, and quality of care) to the utilization of screening services at selected public health centers. The studies were conducted at the same time between July and August of 2022.

### Target population

The study population for the FGDs was women aged 25–49 years visiting the outpatient clinics. For the survey, the target group was women attending cervical cancer screening clinics. The study was conducted in Addis Ababa, the capital of Ethiopia with 11 sub-cities and 126 woredas (districts). There were 90 public health centers actively providing cervical cancer screening services in the city. The metro area population of Addis Ababa was 5,228,000 in 2022 [18].

### Sample size

For the FGDs, a total of 66 women were purposively recruited from the outpatient clinics. The inclusion criteria were women aged 25–49 years and given their consent to participate in the study. Women outside the age group 25–49 were excluded from the study. A total of eight FGDs (two per health center) were done, each having a range of 8–10 participants based on the recommendation of Silverman, 2004 [19]. The discussions lasted for 45–60 min. The sample size for the quantitative utilization survey was calculated using a single population proportion formula, n = z^2^ * p * q / e^2^. Where: n = sample size, z = 1.96 for a confidence level (α) of 95%, p = proportion (0.0547) [15], q = 1-p (0.9453), e = margin of error (5%). Hence, a total sample size (n) of 80 women was determined for the study. The sample was proportionately allocated to the four health centers (20 exit interviews per facility). Women for exit interviews were consecutively recruited (including all accessible subjects) from cervical cancer clinics to reach the sample size of 80. The inclusion criteria were women who received screening and had given their consent to participate in the study. Women who were not screened were excluded from the study.

### Study settings

The qualitative and quantitative data collection was done in four high-patient volume public health centers: Bole 17, Jagama Kello, Kality, and Kolfe Woreda 9 in Addis Ababa. The list was obtained from Addis Ababa City Administration Health Bureau. The cervical cancer clinics were mainly run by midwives or nurses and offered public education, screening, and cervical precancerous lesions treatment services.

### Data collection

Parallel data collection was managed on the different factors influencing the uptake and utilization of cervical cancer services using interview guides. The guides were developed considering the various thematic areas reported by similar previous studies [20–22]. A female research assistant (MPH) experienced in mixed methods research, and fluent in Amharic (the national language) was recruited and trained to facilitate the face-to-face FGDs. There was no relationship established with the participants before the commencement of the study and written informed consent of participants was obtained before discussions/interviews. The sociodemographic characteristics and the views of all participants were collected by probing the key questions until saturation. The questions covered information on the women’s perception of cervical cancer and its screening, personal, financial, and sociocultural barriers to cervical cancer screening uptake, and suggestions. The interviews were held in the health centers’ quiet rooms and the responses were recorded using a voice recorder, and the discussions took from 45 to 60 min. To ensure data quality, the FGDs were reviewed by the principal investigator and the facilitator immediately after completion of the discussions to see the validity of the guides in answering the research questions, and whether the participants’ responses were enabled to draw good conclusions. While the face-to-face exit interviews were conducted in Amharic by four research assistants (BSc in Public Health). The data were collected using an Open Data Kit (ODK) tool installed with a structured questionnaire. The tool was prepared in English and translated into Amharic. Language consistency was established by the translation-retranslation method. The validity of the questionnaire was tested in one health center before commencing the actual data collection and changes were made where necessary. Data were collected on women’s socio-demographic characteristics, perception of cervical cancer and its screening, the decision for screening, referral challenges, waiting times, coordination, preference for and capacity of providers, and improvement area. Each interview was completed within 30 min. The data manager and principal investigator oversaw data collection and consistency daily. Since the flow of clients was low, all women who came from Monday to Friday for cervical cancer screening were interviewed to meet the required sample size of 80.

### Data analysis

The qualitative and quantitative data content analysis was handled separately. The English translations of the eight FGDs were coded and analyzed into five themes identified in advance and seventeen sub-thematic areas using Microsoft Excel by the principal investigator. The similarities and differences of the codes were checked for the various thematic areas including perceptions about cervical cancer and its screening services, personal, financial, and sociocultural barriers to cervical cancer screening, and suggestions for improvement. Exit interview data were cleaned. Descriptive and inferential statistical analyses were carried out using STATA version 17. First, descriptions of the frequency of socio-demographic characteristics of participants, point of decision, perception, coordination, and areas of improvement of the cervical cancer screening services were done. The sociodemographic characteristics and point of decision were independent variables, with the frequency of screening, provider preference, and safety of cervical cancer screening being the dependent variables. The association between independent and dependent variables was analyzed using a logistic regression model. Multivariate logistic regression analysis was done to identify factors associated with the frequency of screening, provider’s preference, and safety of cervical cancer screening services considering only those variables with *p*-values < 0.2. The significance of the variables in the final model was determined using *p*-values < 0.05 and a 95% confidence interval of the adjusted odds ratio (AOR) that did not include unity. The qualitative and quantitative data were integrated for the interpretation of the findings. Finally, the information was presented using tables.

## Results

### Qualitative and quantitative findings

#### Socio-demographic characteristics of the participants

Table 1 gives the socio-demographic characteristics of the FGD participants. The majority, 35 (53%) were aged 30–39, with 53 (80.3%) being married. Among the respondents, 44 (66.7%) were employed, 28 (42.4%) had secondary-level education, and 52 (78.8%) of them had 1–3 children.

**Table 1:** Socio-demographic characteristics of focus group discussion participants (n = 66)

Variables	Participants	Percent
Age group (years		
25–29	10	15.2
30–39	35	53.0
40–49	21	31.8
Marital status		
Married	53	80.3
Single	4	6.1
Divorced	6	9.1
Widowed	3	4.5
Employment status		
Employed/self-employed	44	66.7
Unemployed	22	33.3
Education status		
Primary	21	31.8
Secondary	28	42.4
Tertiary/higher	13	19.7
None	4	6.1
Number of children		
0	8	12.1
1–3	52	78.8
4–6	6	9.1

While for exit interviews, a total of 80 women participated in the study. About 44% of the respondents were aged 30–39, with 61% being married, 50% employed, 38% have completed secondary education, and over half had 1–3 children (Table 2).

**Table 2:** Sociodemographic characteristics of women who participated in exit interviews (n = 80)

Variables	Participants	Percent
Age group (years)		
< 30	12	15.0
30–39	35	43.8
40–49	27	33.8
50+	6	7.5
Marital status		
Married	49	61.3
Single	8	10.0
Separated	3	3.8
Divorced	12	15.0
Widowed	8	10.0
Employment status		
Employed/self-employed	40	50.0
Unemployed	39	48.8
Retired	1	1.3
Education status		
Uneducated	18	22.5
Primary	24	30.0
Secondary	30	37.5
Tertiary/higher	8	10.0
Number of children		
0	13	16.3
1–3	45	56.3
4+	22	27.5

#### Participants’ perceptions

Risk factors: The FGD participants mentioned that uterine viral infection, multiple sexual partners, and early-age sexual practice as key risk factors for acquiring cervical cancer. Smoking, excessive alcohol consumption, and uterine tumor were also mentioned.

Signs and symptoms: Unusual continuous smelling vaginal discharge and itching, irregular and heavy menstruation or bleeding, pain during sexual intercourse, and frequent lower back pain were the major signs and symptoms mentioned by the FGD respondents.

Prevention and screening: The prevention measures mentioned by the FGD participants were precancer screening, avoiding promiscuous sexual practices, and using condoms to avoid exposure to HIV and sexually transmitted infections (STIs). However, one respondent had a wrong perception that eating cabbage and papaya prevents cancer. On screening, the respondents stated that it helps to identify pre-cancer lesions and infections, and prevents death. Whereas the exit interview participants responded that cervical cancer screening was safe (74%) and could prevent death (60%).

#### Personal barriers to the uptake of Cervical cancer screening

The majority of respondents had a lack of awareness of cervical cancer and its screening services, fear of procedure, and embarrassment, followed by a lack of susceptibility to and severity of disease, and fear of screening outcomes.

*“The test is very scary because a device that you do not know enters your cervix. We fear that we may not be able to give birth again. If we had taken the cervical cancer screening training it would facilitate screening. I was married but my husband is dead. So, I do not think I am vulnerable to the disease because I had no relationship afterward. Therefore, I think it is not a must for me.”* (Respondent 1 in FGD 5).

The majority of respondents were not willing to expose their private parts to male providers.

*“In our culture, it is difficult for us to show our reproductive organs except when we give birth or face a problem. I would like to get screened and know my status but not by a male provider. You feel embarrassed when you are checked by a male. You will not be as free as when you are screened by a female. I think that is why most of us are afraid to be screened.”* (Respondent 4 in FGD 1).

The major household barriers reported by the participants were a lack of male partner support and childcare.

***“****If I have pain internally, I do not tell my husband. I have to come and be screened at the health facility. But if you say I am going for screening, you will face obstacles. If you have pain, men may not help you. There is a problem. I know my illness and if I tell my husband about screening, he will not be supportive. I will come to a facility, and be screened on my own.”* (Respondent 7 in FGD 2).

#### Financial barriers to Cervical cancer screening

All participants of the study witnessed that there were no cash payments required for cervical cancer screening services at facilities. However, the Pap smear tests were done at private diagnostics with an out-of-pocket payment of roughly USD10 per test. Some of the FGD respondents mentioned transportation costs and loss of daily wages as challenges. Likewise, for 30% of the exit interview participants, transportation fare was a barrier to screening utilization at facilities.

#### Socio-cultural barriers to the uptake of Cervical cancer screening

Community beliefs and practices including the perception that cancer was contagious and had no cure, and that it was punishment by God were linked to low uptake of screening. Cultural issues such as “yebet tata”/spiritual problems, using traditional medicines, and “holy water” were prevalent. There was no awareness creation in religious institutions. Fear of discrimination resulted in low disclosure of screening plans and/or test outcomes.

*“If you are a cancer patient, someone will point it out to you. If you are diagnosed with cancer, they just give up on you, they consider you as if you are dead. This shortens your life. You are worried about what someone will say about you. The whole family just gets discouraged. If you have cancer, your life is very difficult.”* (Respondent 5 in FGD 8).

#### Referrals, coordination, and quality of care at facilities

Half of the survey participants had self-referred to cervical cancer screening clinics. Women decided to go for cervical cancer screening during the ART clinic attendance (35%), following public awareness messages (28.8%), and family planning visits (25%). Table 3 presents the participants’ views on the level of coordination and quality of care at the facilities. Of the 80 women, 69% had been screened once, 24% twice, and 8% more than twice. The majority (80%) said that the waiting time for screening services in the facility was less than an hour. Forty-five percent (45%) had a preference for a type of provider with 41% saying they preferred being screened by a doctor, and 39% saying they preferred a midwife. The health providers’ capacity was rated excellent by 46% of the women while the overall coordination and quality of care was rated excellent by 36% of the women.

**Table 3:** Women’s report on the coordination and quality of cervical cancer care (n = 80)

Variables	Count	Percent
Frequency of screening for cervical cancer		
Once	55	68.8
Twice	19	23.8
More than twice	6	7.5
Waiting time for the screening service in the facility		
< 1 h	64	80.0
1–2 h	11	13.8
> 2 h	5	6.3
Had a preference for a provider		
Yes	36	45.0
No	44	55.0
The most preferred provider		
Doctor	33	41.3
Midwife	31	38.8
Nurse	15	18.8
Health officer	1	1.3
Rating of the capacity of a health professional		
Poor	0	0.0
Below expectation	4	5.0
Meets expectation	20	25.0
Above expectation	19	23.8
Excellent	37	46.3
Rating of the overall coordination and quality of care		
Poor	4	5.0
Below expectation	6	7.5
Meets expectation	28	35.0
Above expectation	13	16.3
Excellent	29	36.3

#### Factors associated with the utilization of Cervical cancer screening

Table 4 summarizes factors associated with the frequency, provider preference, and safety of cervical cancer screening. Women aged ≥ 40 years were 13.9 times more likely to utilize cervical cancer screening compared to those aged < 30 years (AOR = 13.85; 95% CI: 1.40, 136.74), *p*-value < 0.05. Preference for a female provider was 7.1 times higher (AOR = 7.07; 95% CI: 1.53, 32.75) among women screened after attending antiretroviral therapy clinics, and 6.9 times higher (AOR = 6.87; 95% CI: 1.02, 46.44) for women screened during abnormal vaginal bleeding than those screened after visiting family planning clinics, *p*-value < 0.05. Safety of screening was negatively associated with women aged 30–39 (AOR = 0.045; 95% CI: 0.003, 0.696), and women who attended primary education, and secondary education and above, (AOR = 0.016; 95% CI: 0.001, 0.262), and (AOR = 0.054; 95% CI: 0.004, 0.724), respectively.

**Table 4:** Factors associated with frequency, provider preference, and safety of cervical cancer screening (n = 80)

Variables	Frequency of screening		COR (95% CI)	*p*-value	AOR (95% CI)	*p*-value
	Once n (%)	Twice + n (%)				
Age group (years)						
< 30	11 (91.7)	1 (8.3)				
30–39	31 (88.6)	4 (11.4)	1.42	0.765	1.16 (0.11, 12.48)	0.905
40+	13 (39.4)	20 (60.6)	16.92	0.010	13.85 (1.40, 136.74)	0.024*
Education status						
Uneducated	13 (72.2)	5 (27.8)	1.70	0.428	0.75 (0.16, 3.53)	0.713
Primary	11 (45.8)	13 (54.2)	5.23	0.005	3.11 (0.79, 12.31)	0.105
Secondary and above	31 (81.6)	7 (18.4)				
Constant					0.08	0.016
	Provider preference					
	Yes n (%)	No n (%)				
Current marital status						
Married	33 (67.3)	16 (32.7)	2.98	0.022	2.09 (0.69, 6.34)	0.192
Unmarried	26 (83.9)	5 (16.1)				
Education status						
Uneducated	6 (33.3)	12 (66.7)	3.43	0.041	3.73 (0.92, 15.09)	0.065
Primary	14 (58.3)	10 (41.7)	1.22	0.704	1.01 (0.31, 3.33)	0.989
Secondary	24 (63.2)	14 (36.8)				
Decision for screening						
Family planning	15 (75.0)	5 (25.0)				
Public awareness	15 (65.2)	8 (34.8)	1.75	0.437	1.98 (0.38, 10.39)	0.421
ART clinic attendance	25 (89.3)	3 (10.7)	8.44	0.002	7.07 (1.53, 32.75)	0.012*
Other	4 (44.4)	5 (55.6)	8.00	0.021	6.87 (1.02, 46.44)	0.048*
Constant					0.15	0.004
	Safety of screening					
	Yes n (%)	No n (%)				
Age group (years)						
< 30	11 (91.7)	1 (8.3)				
30–39	21 (60.0)	14 (40.0)	0.14	0.070	0.045 (0.003, 0.696)	0.026*
40+	27 (81.8)	6 (18.2)	0.41	0.432	0.2 (0.012, 3.431)	0.267
Current marital status						
Married	33 (67.3)	16 (32.7)	2.52	0.108	0.247 (0.055, 1.115)	0.069
Unmarried	26 (83.9)	5 (16.1)				
Education status						
Uneducated	17 (94.4)	1 (5.6)				
Primary	14 (58.3)	10 (41.7)	0.08	0.024	0.016 (0.001, 0.262)	0.004*
Secondary and above	28 (73.7)	10 (26.3)	0.16	0.099	0.054 (0.004, 0.724)	0.028*
The decision for cervical cancer screening						
Public awareness	15 (65.2)	8 (34.8)				
Family planning	15 (75.0)	5 (25.0)	1.60	0.488	0.702 (0.125, 3.949)	0.688
ART clinic attendance	25 (89.3)	3 (10.7)	4.44	0.047	3.778 (0.647, 22.059)	0.140
Other	4 (44.4)	5 (565.6)	0.43	0.288	0.157 (0.018, 1.372)	0.094
Constant					1413.02	0.002

COR: Crude Odds Ratio; AOR: Adjusted Odds Ratio. *Significantly associated factors

#### Suggestions for improving Cervical cancer screening

The key suggestions by FGD participants for improving cervical cancer screening included strengthening the dissemination of public awareness messages through radio and TV, at festivals and holidays and traditional burial societies (edirs), and door-to-door visits by health extension workers (HEWs). Those who received awareness messages share them with others including about free screening services. As facilities provide regular health education, effort should concurrently go towards strengthening the single visit approach (SVA), integrated services, reducing waiting times, and compassionate services by trained health workers, and arranging for free referral tests.

*“When I often come to the health center in the morning, many people gather at the facility. There was a TV but it has never been on. Firstly, it is good for health facilities to disseminate cervical cancer messages in the morning or afternoon when more clients show up. When something is said over and over again it comes to your mind. Secondly, if there is a way to work with religious institutions it could encourage women to go for cervical cancer screening. Thirdly, mothers should create awareness among their children as youth play the biggest role in the fight against cancer. Finally, a woman can also reach out to two or three of her friends.”* (Respondent 7 in FGD 4).

Moreover, the exit interviews indicated that nearly half of the respondents (49%) demanded shortening waiting time, 44% recommended the provision of transportation allowance, and 40% proposed improving the staff attitude.

## Discussion

This facility-based study examined factors influencing the uptake (qualitative) and utilization (quantitative) of cervical cancer screening services focusing on personal, financial, and sociocultural barriers, providers’ capacity, coordination and quality of care, and patient-centered care at primary healthcare facilities in the metropolitan city of Addis Ababa. In contrast, other Ethiopian studies identified and addressed the barriers and/or facilitators for cervical cancer uptake or utilization in rural or urban settings mainly using community-based cross-sectional studies targeting women of reproductive age. The study findings are aimed to be linked with our other studies on the national cancer control plan strategies, and health system factors influencing cervical cancer services at primary, secondary, and tertiary levels to come up with a comprehensive quality improvement measure. The majority of FGDs respondents had no clear understanding of cervical cancer and its screening services. The major barriers to the uptake of screening were fear of procedure, embarrassment, gender of providers, lack of support from partners, and childcare. The exit interviews revealed that nearly 89% of women decided to go for screening in health facilities. The screening was safe for 59 (73.8%) women, and an overall waiting time at the facility was < 1 h for 64 (80%) participants, and there was no provider preference for 44 (55%) of them. The capacity of providers and overall coordination was excellent for 37 (46.3%) and 29 (36.3%) of women, respectively. For improving screening, the FGD participants suggested intensifying public awareness messages, while among the exit interview participants 39 (48.8%) of them proposed shortening waiting time, 35 (43.8%) of them demanded transportation allowance, and 32 (40%) of them required changing the staff attitude. The study provides feasible actions and recommendations to improve the uptake and utilization of cervical cancer screening which paves the road to meeting the WHO 90-70-90 targets by 2030 through integrated contributions made by decision-makers, providers, communities, and service users.

In Ethiopia, exploring factors affecting the uptake of cervical cancer screening through FGDs was very limited, and inadequately documented. Our FGDs pointed to gaps in understanding the causes, risk factors, prevention mechanisms, screening, and treatment of cervical cancer though some of the respondents believed that screening identifies pre-cancer lesions and infections, and prevents death. Similar gaps were reported by various Ethiopian and Zimbabwean studies [20–23]. One of our FGD respondents had a wrong perception such as eating cabbage and papaya prevents cancer. A study conducted in the Sidama region, south-central part of Ethiopia, revealed wrong perceptions such as poor hygiene, trauma, and urinating in the sun cause cervical cancer [23]. The modifying factors that affect women’s perceptions, knowledge, socioeconomic, and personality factors may influence the individual beliefs of women which in turn affect their behaviors and cues to action. Perceived threat results from understanding perceived susceptibility to and severity of disease [24, 25].

Our FGDs showed that women aged ≥ 40 years were 13.9 times more likely to utilize cervical cancer screening than those aged < 30 years probably due to their repeated exposure to public awareness messages during health facility visits for consultation of various providers in ART clinics, family planning, and outpatient clinics. This finding is supported by previous Ethiopian studies that showed older age was strongly associated with the uptake and utilization of cervical cancer screening among age-eligible women [26–28]. Moreover, a South African study showed that women aged 35–44 years had a higher uptake of screening with Pap smear tests [29]. The other barriers to the screening uptake were a lack of awareness of cervical cancer and its screening services, fear of procedure or pain, embarrassment, perceived lack of susceptibility to and severity of disease, and fear of screening outcomes. These findings agree with several previous studies [26, 27, 30–33]. Our study identified inadequate public education about cervical cancer. This relates to an Ethiopian study that showed only 27.7% of women had adequate knowledge of cervical cancer screening [34]. Lack of awareness was the major factor that contributed to lower cervical cancer screening utilization of 10.3% [35], and 5.47% [15] in Ethiopia. Similarly, an Indian study reported only 5% of 30–60 years old women underwent Pap smear test screening during their lifetime [36] and 40.7% of the women in Bahrain [37]. Our findings showed that 74% of FGD participants and 68% of the exit interview participants had attained at least a primary school education. Existing evidence suggests that educated women are likely to have a better understanding, self-efficacy, and cues to cervical cancer screening [15, 35]. This was found to be a good facilitator for promoting the uptake and utilization of the screening services. Half of the women in the utilization study were self-referred to screening clinics which shows a need for strengthening the provider’s referral services. The majority of women decided to go for cervical cancer screening at the ART clinic, from public awareness messaging and family planning visits, suggesting that one-on-one counseling and group education could be effective mechanisms for disseminating cervical cancer messages. This finding was supported by other Ethiopian studies [15, 35].

Although cervical cancer screening was free, the transportation cost was mentioned as a challenge by the FGD and exit interview participants, more so, when screening was done outside of the health facility or women were referred for specialty care. Private diagnostic facilities charged roughly USD 10 per Pap smear test in Addis Ababa. A busy work schedule was also a challenge to some of the respondents. This has also been reported elsewhere [22, 31, 33].

The majority of utilization study participants did not have a preference for the provider. Preference for a provider was 7 times higher among women screened after attending antiretroviral therapy clinics, and women screened during abnormal vaginal bleeding. The provider preference issues were reported by studies from Ghana [31, 32]. Similarly, a study conducted at primary healthcare centers in Bahrain showed that 83.3% of participants felt embarrassed when examined by a male doctor [37]. The other health facility-related challenges identified by FGDs were long waiting times, fear of acquiring infection from the screening device/speculum, lack of screening device options, and doubting the capacity of providers. The safety of screening was negatively associated with women aged 30–39, and those who attended primary, and secondary education and above. The health system challenges were reported by several previous studies including lack of infrastructure, the attitude of healthcare providers, lack of privacy, misdiagnosis, and others [22, 30, 32, 33].

The FGD respondents reported that the major societal acts were discouraging screening, propagating that cancer is contagious and has no cure, and it is an act against God. Similar issues such as poor hygiene, the devil’s intrusion, God’s punishment, and belief in no cure for cervical cancer were reported by other Ethiopian studies [21, 23]. Cultural issues such as “yebet tata”/ spiritual problems, traditional medicines treatment, and “holy water” were prevalent in the community. Moreover, awareness creation on cancer was uncommon in religious institutions. Due to fear of discrimination or stigma, people did not want to disclose their screening plans or the outcomes of the test. These agree with the findings of various African studies [22, 24, 32].

Study participants suggested that improving the uptake and utilization of cervical cancer screening services required the dissemination of public awareness messages in media (radio and TV), at festivals and holidays, and in traditional burial societies (edirs). This was supported by an Ethiopian study that reported on the importance of demand creation using mass media [30]. Moreover, regular health education at facilities, door-to-door services by health extension workers (HEWs), and transmission of messages to fellow women in the community were recommended. Shortening waiting time, allocating transportation allowance, improving the staff attitude and capacity, provision of integrated single visit approach (SVA) services, and arranging free referral tests were other areas to improve on. Studies reported that training of providers, application of incentives for screening services, coordination, and collaboration are required for strengthening the health system [35, 38]. A similar recommendation was put forward by a global cancer control study that focused on responding to the growing burden, rising costs, and inequalities in access to cancer care [39]. Additionally, a study in the Southern part of Ethiopia showed that the implementation of an appropriate awareness-creation method and linkage with sexually transmitted infection (STI) services were necessary for improving the utilization of cervical cancer screening services [35]. Furthermore, a study conducted in Addis Ababa reported an increase in cervical cancer screening uptake through the provision of one-on-one health education supported with printed educational materials [40]. To address the misconceptions about female cancers at the community level, an Ethiopian study put forward the importance of designing social and behavioral change strategies [23]. Most importantly, the recommendations of the WHO Cancer Resolution of 2017 must be implemented at the national level to reduce the cancer burden, avoid unnecessary suffering, and save as many lives as possible [1].

### Limitations of the study

One of the limitations of the study was due to a lack of resources it was not possible to consider low-volume health centers to understand factors influencing the uptake and utilization of cervical cancer screening. This could have given a chance to see if uptake and utilization were influenced by the volume of services. Secondly, the knowledge of providers about cervical cancer, its prevention and screening services, and the rate of uptake and utilization was not measured in the selected sites. Thirdly, a lack of generalizability and social desirability bias were gaps in the study as it was conducted in the capital city (Addis Ababa) only mainly due to resources constraint. Future studies may consider rural settings and other regions in the country, and an assessment of the competency of cervical cancer service providers in facilities.

## Conclusions

The study has identified low perception of women (causes, risk factors, prevention mechanisms, screening and treatment of cervical cancer), and the main barriers to cervical cancer screening included personal (lack of knowledge, inadequate awareness, embarrassment, fear of procedure and outcome of test, and lack of susceptibility to and severity of disease), financial (transportation incentives for the referral of the most at risk women to primary care, and free referral testing), socio-cultural (discrimination, absence of male support, cultural and religious beliefs), health system (delays at facilities, provider preference issues, the capacity and training of providers, adequacy of infrastructure and space, and lack of screening device options and privacy), and cross-cutting (coordination and communication). An uptake and utilization improvement strategy incorporating these factors is recommended such as improved dissemination of public awareness messages through media (radio and TV programs), in the community, among religious leaders, traditional healers, cervical cancer survivors, and at public gatherings. The primary healthcare and health extension workers are required to be trained and continue providing compassionate, respectful and caring, coordinated, and integrated services with shortened waiting times in the facility. The health system should strive to meet the preferences, needs, and values of women, and minimize physical, financial, and emotional strain on cervical cancer patients and their families.

### Electronic supplementary material

Below is the link to the electronic supplementary material.

**Additional file 1:** Guidelines for Assessment of Factors Influencing Utilization of Cervical Cancer Screening Services

**Additional file 2:** Guidelines for Exploring Factors Affecting the Uptake of Cervical Cancer Screening Services

## Data Availability

All the analyzed qualitative and quantitative datasets are available from the corresponding author (KH) upon request.

## Abbreviations

AOR: Adjusted odds ratio
ASIR: Age-standardized incidence rate
COR: Crude odds ratio
FGD: Focus Group Discussion
HEWs: Health extension workers
HPV: Human papillomavirus
LMICs: Low-and medium-income countries
MoH: Ministry of Health
NCCP: National Cancer Control Plan
NCDs: Non-communicable diseases
SSA: Sub-Saharan Africa
STA: Screen-and-treat approach
STIs: Sexually transmitted infections
SVA: Single-visit approach
VIA: Visual inspection with acetic acid
WHO: World Health Organization
