# Supplementary material for: Factors influencing the uptake and utilization of cervical cancer screening services among women attending public health centers in Addis Ababa, Ethiopia: mixed methods study
Source: BMC Womens Health. 2024 Jan 2;24:3. doi: 10.1186/s12905-023-02850-x (PMC10763437; doi:10.1186/s12905-023-02850-x)
Supplement: Supplementary file 1 — Additional file 1: Guidelines for Assessment of Factors Influencing Utilization of Cervical Cancer Screening Services [file 12905_2023_2850_MOESM1_ESM.docx]

**Additional file 1. Guidelines for Assessment of Factors Influencing Utilization of Cervical Cancer Screening Services.**

**INTERVIEWER:** Please explain the following questions to participants to assess the utilization of cervical cancer screening services.

1. **Socio-demographic characteristics of participants.**

| **I. Age group (years)** | **# of participants** |
| --- | --- |
| a. 25-29 |  |
| b. 30–39 |  |
| c. 40–49 |  |
| d. 50+ |  |
| **II. Marital status** |  |
| a. Married |  |
| b. Single |  |
| c. Separated |  |
| d. Divorced |  |
| e. Widowed |  |
| **III. Employment status** |  |
| a. Employed/self-employed |  |
| b. Unemployed |  |
| c. Retired |  |
| **IV. Education status** |  |
| a. Primary |  |
| b. Secondary |  |
| c. Tertiary/higher |  |
| d. None |  |
| **V. Number of children** |  |
| a. 1–3 |  |
| b. 4-6 |  |
| c. 7+ |  |

1. **The key questions below will examine the demand-side factors affecting the utilization of cervical cancer screening services.**

| **I. Decision for screening** |  | |  |
| --- | --- | --- | --- |
| Why did you decide to go for cervical cancer screening at the facility? | a. Public awareness | | 1 |
|  | b. Outpatient clinic visit | | 2 |
|  | c. ART clinic attendance | | 3 |
|  | d. Abnormal vaginal bleeding | | 4 |
|  | e. Increased vaginal discharge | | 5 |
| **II. Perception** |  | |  |
| What is your perception of early cervical cancer screening services? | a. Safe | | 1 |
|  | b. Cost effective | | 2 |
|  | c. Prevents death from the disease | | 3 |
| **III. Referral system** |  | |  |
| (a) Who has referred you to the facility? | a. Self | | 1 |
|  | b. Health extension worker (HEW) | | 2 |
|  | c. Health development army (HDA) | | 3 |
|  | d. Third party | | 4 |
| (b) What are the challenges in the linkage of women with primary healthcare facilities? | a. Distance to the facility | | 1 |
|  | b. Transportation cost | | 2 |
|  | c. Referral forms | | 3 |
|  | d. Other | | 4 |
| **IV. Coordination and quality of care** |  | |  |
| (a) How many times have you been screened for cervical cancer? | a. Once | | 1 |
|  | b. Twice | | 2 |
|  | c. More than twice | | 3 |
| (b) How long did you wait for the screening service in the facility? | a. < 1 hour | | 1 |
|  | b. 1-2 hours | | 2 |
|  | c. > 2 hours | | 3 |
| (c) Did you have a preference for a provider? | Yes | No |  |
|  | 1 | 2 |  |
| (d) How would you rate the capacity of health professionals in the facility? | a. Poor | | 1 |
|  | b. Below expectation | | 2 |
|  | c. Meet expectation | | 3 |
|  | d. Above expectation | | 4 |
|  | e. Excellent | | 5 |
| (e) How would you rate the overall coordination and quality of care in the facility? | a. Poor | | 1 |
|  | b. Below expectation | | 2 |
|  | c. Meet expectation | | 3 |
|  | d. Above expectation | | 4 |
|  | e. Excellent | | 5 |
| **V. Key areas for improvement** |  | |  |
| Which of the following areas are key for better utilization of cervical cancer screening services at the facility? | a. Provide transportation allowance | | 1 |
|  | b. Shorten the waiting time | | 2 |
|  | c. Improve staff attitude/capacity | | 3 |
|  | d. Preference for a provider | | 4 |
|  | e. Spouse or partner support | | 5 |
|  | f. Other | | 6 |
