# Supplementary material for: Factors influencing the uptake and utilization of cervical cancer screening services among women attending public health centers in Addis Ababa, Ethiopia: mixed methods study
Source: BMC Womens Health. 2024 Jan 2;24:3. doi: 10.1186/s12905-023-02850-x (PMC10763437; doi:10.1186/s12905-023-02850-x)
Supplement: Supplementary file 2 — Additional file 2: Guidelines for Exploring Factors Affecting the Uptake of Cervical Cancer Screening Services [file 12905_2023_2850_MOESM2_ESM.docx]

**Additional file 2. Guidelines for Exploring Factors Affecting the Uptake of Cervical Cancer Screening Services.**

**FACILITATOR:** Good morning/afternoon. Introduce yourself and ask the focus group discussion (FGD) participants to introduce themselves. Ensure that the consent form is signed by individual participants. Then explain the following questions that aim to explore factors influencing cervical cancer screening with women who visit the outpatient clinic.

1. Socio-demographic characteristics of participants/mothers

| **Characteristic** | **The number of participants** |
| --- | --- |
| 1. **Age group (years)** |  |
| 25-29 |  |
| 30–39 |  |
| 40–49 |  |
| 50+ |  |
| 1. **Marital status** |  |
| Married |  |
| Single |  |
| Separated |  |
| Divorced |  |
| Widowed |  |
| 1. **Employment status** |  |
| Employed/self-employed |  |
| Unemployed |  |
| Retired |  |
| 1. **Education status** |  |
| Primary |  |
| Secondary |  |
| Tertiary/higher |  |
| None |  |
| 1. **Number of children** |  |
| 1–3 |  |
| 4-6 |  |
| 7+ |  |

1. The 6 key questions below will lead the focus group discussions (FGDs) on factors affecting the uptake of cervical cancer screening services at public health centers.

**Purpose of FGD**

The purpose of this FGD is to explore in-depth, demand-side factors influencing the uptake of quality cervical cancer screening services among women who visit outpatient clinics. The study intends to specifically elucidate the following:

1. Women’s perception or awareness about cervical cancer and its screening service.

2. Personal, financial, and sociocultural factors affecting the uptake of cervical cancer screening services.

3. Suggestions for improving the uptake of cervical cancer screening services.

**FGD procedures**

My name is________. Thanks a lot for joining this important discussion session. I would like to go through a few important points before we begin the discussion. The interview will last approximately 45-60 minutes. The study is approved by Strathmore University Institutional Scientific and Ethical Review Committee (SU-ISERC) and Addis Ababa City Administration Health Bureau. Everything we discuss during this interview is strictly confidential and your real name will not be revealed in any of our findings. Hence, kindly try to be frank and truthful when you are responding to the FGD questions. I will provide you a consent form to read and sign if you find it acceptable with you. To capture accurate information, this interview will be recorded using a smartphone or voice recorder. If consent is given by the participants, the facilitator will instruct the notetaker to commence tape recording.

**Focus Group Discussion Guide**

| **Key questions** | **Probes** |
| --- | --- |
| 1. **Perception or awareness**   What is your **perception or awareness** of cervical cancer and its screening services? | 1. What risk factors can you mention?  2. What signs and symptoms are you aware of?  3. What preventive measures do you know?  4. What is your understanding of the screening? |
| 1. **Personal barriers**   What are the **personal barriers** to the uptake of cervical cancer screening services? | 1. What is the influence of education, lack of knowledge of screening facilities, language, and marital status/childcare on the uptake of cervical cancer screening?  2. What is the impact of a perceived lack of susceptibility to and severity of disease, feeling of embarrassment, fear of the procedure/pain, and preference for the gender of the screener on the uptake of cervical cancer screening? |
| 1. **Financial barriers**   What are the **financial barriers** to the uptake of cervical cancer screening services? | 1. What is the influence of out-of-pocket payment, transportation, and lost wages on pursuing cervical cancer screening? |
| 1. **Sociocultural barriers**   What are the **socio-cultural barriers** to the uptake of cervical cancer screening services? | 1. What are the myths/misconceptions about cervical cancer screening?  2. What kind of stigma/discrimination did you encounter while seeking cervical cancer screening?  3. What are the views of different religious beliefs on cervical cancer screening? |
| 1. **Suggestions**   What are your **suggestions** for improving the cervical cancer screening uptake by primary healthcare facilities? | 1. What do you think are the best ways to improve public awareness, transportation, and referral networks in cervical cancer screening? |
| 1. **Other factors**   Are there any **other factors** you would like to mention about the uptake of cervical cancer screening? |  |
